# *Rickettsia amblyommii* infecting *Amblyomma sculptum* in endemic spotted fever area from southeastern Brazil

**DOI:** 10.1590/0074-02760150266

**Published:** 2015-12

**Authors:** Emília de Carvalho Nunes, Vinicius Figueiredo Vizzoni, Daniel Leal Navarro, Felipe Campos de Melo Iani, Liliane Silva Durães, Erik Daemon, Carlos Augusto Gomes Soares, Gilberto Salles Gazeta

**Affiliations:** 1 Universidade Federal de Juiz de Fora, Instituto de Ciências Biológicas, Programa de Pós-Graduação em Ciências Biológicas, Comportamento e Biologia Animal, Juiz de Fora, MG, Brasil; 2 Fundação Oswaldo Cruz, Instituto Oswaldo Cruz, Laboratório de Referência Nacional em Vetores das Riquetsioses, Rio de Janeiro, RJ, Brasil; 3 Universidade Federal do Rio de Janeiro, Instituto de Biologia, Departamento de Genética, Laboratório de Genética Molecular de Eucariontes e Simbiontes, Rio de Janeiro, RJ, Brasil; 4 Fundação Ezequiel Dias, Laboratório Central de Minas Gerais, Serviço deVirologia e Riquetsioses, Belo Horizonte, MG, Brasil

**Keywords:** Rickettsia amblyommii, Amblyomma sculptum, southeastern Brazil -, ticks

## Abstract

The *Rickettsia* bacteria include the aetiological agents for the human spotted fever (SF) disease. In the present study, a SF group*Rickettsia amblyommii* related bacterium was detected in a field collected *Amblyomma sculptum* (*Amblyomma cajennense* species complex) tick from a Brazilian SF endemic site in southeastern Brazil, in the municipality of Juiz de Fora, state of Minas Gerais. Genetic analysis based on genes *ompA*,*ompB* and *htrA* showed that the detected strain, named *R. amblyommii* str. JF, is related to the species*R. amblyommii*.

Over the past 14 years, the number of rickettsial species identified in South America increased from three to more than 10. Initially, only occurrences of *Rickettsia prowazekii*, *Rickettsia typhi*, and *Rickettsia rickettsii* were historically reported, followed by most recent detection of*Rickettsia felis*, *Rickettsia parkeri*,*Rickettsia belli*, *Rickettsia massiliae*,*Rickettsia rhipicephali*, and *Rickettsia amblyommii*from different environmental samples (Labruna 2009).

Among those cited, six species belong to the spotted fever group (SFG), including the known human pathogens *R. rickettsii*, *R. felis*,*R. Parkeri*, and *R. massiliae*, each causing specific rickettsiosis, whereas *R. rhipicephali* and *R. amblyommii* are classified as with still unknown/unclear pathogenicity (Merhej et al. 2014).

*R. rickettsii* is the aetiologic agent of the Rocky Mountain SF, the most severe of all tick-borne rickettsiosis (Parola et al. 2005). In Brazil this species causes the Brazilian SF (BSF), a disease that in the last 14 years was reported in 1,421 cases throughout Brazil, according to official data of the Information System on Notifiable Diseases (dtr2004.saude.gov.br/sinanweb/). During the period of 1995-2004, there were 334 laboratory-confirmed cases of BSF with a 31% lethality rate in the Southeast Region of Brazil. Additional 128 cases, with lethality of 29%, were confirmed from 2005-2007 only in the state of São Paulo (Labruna 2009). In the state of Minas Gerais other BSF cases were also confirmed and in the endemic area of the city of Juiz de Fora, 24 cases were actually confirmed between 2001-2014 (dtr2004.saude.gov.br/sinanweb/). According to Pacheco et al. (2011), 17 cases were notified between 1995-2008, with a lethality of 29%.

Ticks are the most important vectors for SF transmission. In South America, the tick*Amblyomma cajen- nense* has been considered to be the most frequent vector related with SF cases. Interestingly recent genetic and morphological/microscopic analyses showed evidence that *A. cajennense* in fact represents a complex grouping six tick species (*A. cajennensesensu lato* or*s.l.*) (Beati et al. 2013,Nava et al. 2014).

The endemicity of BSF leads Juiz de Fora Health Office to promote a constant environmental monitoring of ticks. Forty-eight horses fed *A. sculptum*ticks were obtained during this vigilance and the specimens were identified according to the new description of species that belong to the *A. cajennense* complex (Nava et al. 2014). The neighbourhoods known as Previdenciários and Monte Castelo were visited and both areas had confirmed human BSF cases. Sampled ticks were processed for molecular analysis and initially submitted to DNA extraction as described elsewhere [method with NaCl (Aljanabi & Martinez 1997)].

*Rickettsia* infected ticks were identified by polymerase chain reaction (PCR) screening for the rickettsial *ompA*, *ompB* and*htrA* genes in 25 μL conventional PCR reactions under the temperature/time cycle: 94ºC 3 min and 30 s (94ºC 30 s, 55ºC 30 s, 72ºC 1 min/Kb] 40X, 72ºC 7 min, 20ºC ∞. *R. parkeri* str. AT#24 DNA was used as a positive control. The primers used were Rr190.70F and Rr190.602R (Regnery et al. 1991) for *ompA* gene, Rr1175F and Rr2608R (Blair et al. 2004) for *htrA*gene and ompB3064-F (5’ggtatagccggaataggttttgacg, present study) and ompB4271-R (5’tcagttttagtgataccgatagcagc, present study) for *ompB* gene. PCR products were purified using HiYield^TM^ Gel/PCR DNA Mini Kit according to manufacturer (Real Genomics^TM^, New Zealand), sequenced in both directions on an automated ABI 3130xl Genetic Analyser (Applied Biosystems^®^, USA) and using the same primers applied for the initial PCR amplifications. Sequence edition was performed with Lasergene software packages (DNASTAR, USA).

One *A. sculptum* female was positive for *Rickettsia*infection as determined by the PCR tests. PCR amplicons of 512 bp, 1,208 bp, and 434 bp were obtained using the primer sets for *ompA*, *ompB* and*htrA*, respectively.

A phylogenetic tree was constructed with concatenated *ompA*,*ompB* and *htrA* sequences, neighbour-joining methods [MEGA 5.2 (Tamura et al. 2011)], and Kimura two-parameter model to estimate genetic divergence (Kimura 1980) and bootstrap values were obtained from 1,000 randomly generated trees. The resulting tree showed that the presently identified strain, here named *R.amblyommii* str. JF, is most closely related to *R. amblyommii* (Figure).

**Figure:**
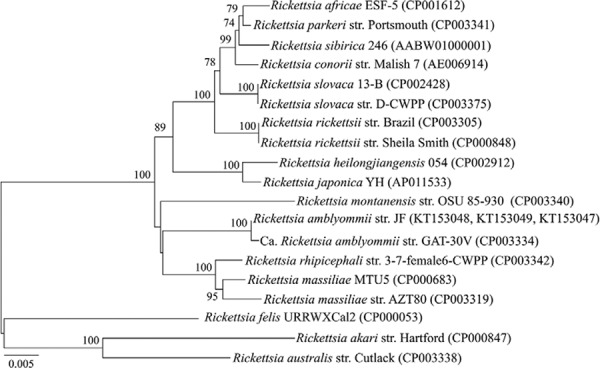
Phylogenetic tree of concatenated spotted fever group rickettsiae*ompA*, *ompB* and *htrA*genes constructed by neighbour-joining method with Kimura two-parameter as evolution model and based on the nucleotide sequences. The GenBank accession codes are presented in parenthesis. The numbers at nodes are the bootstrap values obtained from 1,000 re-samplings. Bootstrap values bellow 70% are not present.

The first detection of *R. amblyommii* occurred after analysis of*Amblyomma americanum* ticks in the United States of America (Burgdorfer et al. 1981). After that, *R. amblyommii* and other genetically related species were found in a wide variety of tick species in different countries in the New World (Labruna et al. 2007, Zanettii et al. 2008, Hun et al. 2011, Saraiva et al. 2013). In the present study, a*R. amblyommii* related infection was characterised in an *A. sculptum* sample, an *A. cajennense*-complex species abundantly observed in Juiz de Fora. Other bacteria genetically related to *R. amblyommii* was previously found associated with *A. cajennenses.l*. from Brazil (Labruna et al. 2004, Soares et al. 2015) and Alves et al. (2014) provided the first description of *R. amblyommii*-like infection in *A. sculptum*tick.

Despite the amount of studies on *Rickettsia* performed in thousands of ticks sampled in southeastern Brazil (Guedes et al. 2005, Gehrke et al. 2009, Pacheco et al. 2009), the present paper is the first report of a *R. amblyommii* related SFG infecting *A. sculptum* in this region.

Several reports indicate that *R. amblyommii* is commonly found in ticks parasitising human (Jiang et al. 2010, Lee et al. 2014) and the observed *R. amblyommii* in Juiz de Fora could distinctly represent an unusual rickettsiosis agent. Primarily nonpathogenic *Rickettsia* is able to cause disease under some circumstances, as reported for *R. parkeri*(Paddock et al. 2004). The pathogenic potential of *R. amblyommii* and genotypically similar strains is still speculative, but increasingly studies have associate these bacteria to rickettsiosis cases (Taylor et al. 1985, Yevich et al. 1995, Billeter et al. 2007, Apperson et al. 2008).

The potential pathogenic capacity of *R. amblyommii* would bring major concerns in terms of public health since this bacterium can infect a variety of vertebrate hosts with wide distribution, including species with dense populations and commonly found in parks or recreational areas (Blanton et al. 2014).

Despite that not all ticks can actively feed and parasitise humans, serological data obtained from dogs and horses showed the circulation of *R. amblyommii*in domestic animals (Barrett et al. 2014). Frequent infestation of these animals with specific tick species could facilitate the transmission of this bacterium to humans. Indeed, ticks frequently found on dogs and horses have been reported to be able to support *R.amblyommii* infection (Bermúdez et al. 2009, Eremeeva et al. 2009) and this bacterium seems to be well adapted to its hosts, presenting highly successful transmission rates (Burgdorfer et al. 1981, Saraiva et al. 2013)*.*

Taking together, these observations suggest that this bacterium may be involved in cases of atypical SF cases and show the need for further studies to elucidate its real pathogenic potential. Although the *R. amblyommii* pathogenic status remains unclear, the present study brings new contribution to the understanding of its complex vector/host network.
